# Moderating effects of affective interventions on manic symptoms and quality of life: A cognitive improvement study based on patients with bipolar disorder

**DOI:** 10.1097/MD.0000000000046027

**Published:** 2025-11-28

**Authors:** Bingwei Zhang, Chao Li, Suilin Jia, Guangdong Chen

**Affiliations:** a Behavioral Medicine Department, Wenzhou Seventh People’s Hospital, Wenzhou, Zhejiang, China; b Psychiatric Complex One Ward, Wenzhou Seventh People’s Hospital, Wenzhou, Zhejiang, China.

**Keywords:** affective interventions, bipolar disorder, cognitive functioning, mania, quality of life

## Abstract

**Background:**

Initially, psychotherapy was the primary treatment for bipolar disorder. However, with advances in biomedical research and the rapid efficacy of medications, psychosocial treatments have been increasingly overlooked. Despite evidence supporting bipolar disorder as a “biological disease,” psychotherapy remains essential as an adjunct to medication and other treatments, including emotional interventions.

**Methods:**

This study included 76 patients with bipolar affective disorder admitted to our hospital between February 2022 and February 2023. Patients were randomly assigned to an intervention group (n = 38) or a control group (n = 38). The control group received conventional treatment, while the intervention group received a 4-week emotional intervention in addition to the conventional treatment. We assessed manic symptoms, quality of life, and cognitive function using the Bech-Rafaelsen Mania Scale, the Short Form-36 Health Survey, and the Insight and Treatment Attitudes Questionnaire before and after the intervention.

**Results:**

There were no significant differences in baseline data between the 2 groups (*P* > .05). After the intervention, Bech-Rafaelsen Mania Scale scores were significantly lower, and Short Form-36 Health Survey and Insight and Treatment Attitudes Questionnaire scores were significantly higher in the intervention group compared to the control group (*P* < .05), indicating an improvement in manic symptoms, quality of life, and cognitive function.

**Conclusion:**

Emotional interventions significantly improve manic symptoms, quality of life, and cognitive function in patients with bipolar disorder. This study supports the integration of emotional interventions into treatment programs to enhance clinical outcomes for these patients.

## 1. Introduction

Bipolar Affective Disorder (BAD) is a common and complex psychiatric disorder characterized by alternating episodes of depressive and manic symptoms, which severely affects patients’ cognitive function and quality of life.^[1]^ According to the latest statistics from the World Health Organization,^[2]^ approximately 2.4% of the global population is affected by bipolar disorder, with a significant increase in the prevalence and recurrence of manic symptoms. Wang et al^[3,4]^ showed that during manic episodes, patients have reduced impulse control and highly excitable behavior, leading to social functioning (SF) and quality of life. arousal, resulting in significantly lower SF and quality of life.

Emotional interventions, as a nonpharmacological treatment, have received extensive attention in recent years in modulating manic symptoms and improving quality of life.^[3,5]^ Affective intervention includes various methods such as cognitive-behavioral therapy, Mindfulness Therapy and Interpersonal and Social Rhythm Therapy (IPSRT). It significantly reduces the frequency and intensity of manic symptoms by adjusting the patient’s emotional and behavioral patterns and improving their emotion regulation.^[6–8]^ Michel et al^[9]^ showed that affective interventions have significant effects in improving the emotional stability of patients with bipolar disorder. At the neurobiological level, affective interventions can improve emotion regulation by modulating neural activity in the brain’s emotion processing centers such as the amygdala, prefrontal cortex, and hippocampus.^[10]^ Meanwhile, affective interventions further enhance patients’ emotional expression by enhancing the plasticity of the brain’s neural networks and facilitating the connectivity and functional integration between neurons.^[11]^ However, the specific mechanisms and long-term effects of affective interventions in patients with bipolar disorder have not been fully clarified, especially in improving cognitive functions, and further in-depth research and validation are still needed. This study aimed to investigate the moderating effect of affective intervention on manic symptoms and quality of life in patients with bipolar disorder, focusing on analyzing the improvement of cognitive function in patients with affective intervention. Through systematic affective intervention for bipolar disorder patients, changes in emotional stability, SF and overall quality of life were assessed, providing scientific basis and practical guidance for clinical treatment.

## 2. Methods

### 2.1. Research subjects

A total of 76 cases of bipolar disorder patients admitted to our hospital from February 2022 to February 2023 were selected as the research subjects, and using the random number table method, according to the patient’s admission time number to expand the grouping, they were divided into the intervention group and the control group, with 38 cases in each group.

#### 2.1.1. Inclusion criteria:

Diagnosis of bipolar disorder, currently in a manic episode, with diagnostic criteria in accordance with the relevant criteria in the Diagnostic and Statistical Manual of Mental Disorders, Fifth Edition^[12]^ (DSM-5) and the International Classification of Diseases, Eleventh Edition^[13]^ (ICD-11); age of 18 to 65 years old; for at least 4 weeks prior to the study, the patient’s medication regimen should have remain stable and no substitution of major psychiatric drugs has been made; patients and their guardians are required to sign an informed consent form, explicitly agreeing to participate in this study and follow the relevant experimental procedures.

#### 2.1.2. Exclusion criteria:

Comorbidity with other severe mental disorders such as schizophrenia, major depressive disorder, borderline personality disorder, etc; presence of severe physical illness or dysfunction; worsening of acute psychiatric symptoms or risk of suicide within 2 weeks prior to enrollment; presence of severe cognitive impairment or mental retardation, unable to cooperate in completing cognitive assessment or affective intervention therapy; pregnant women or breastfeeding women; unable to participate in regular follow-up and assessment due to geography, transportation or other objective reasons. The study was approved by the Ethics Committee of our hospital.

### 2.2. Methodology

The control group was given conventional drug treatment, using lithium carbonate tablets (manufacturer: Beijing Shuanglu Pharmaceutical Co., Ltd, State Drug License H10940002, specification 300 mg), 0.6g per day, oral in divided doses, for 4 weeks; quetiapine tablets (manufacturer: Shanghai Fudan Fuhua Pharmaceutical Co., Ltd, State Drug License H20030012, specification 25 mg), with an initial dose of 50 mg per day, gradually increased to 300mg, divided oral, the course of treatment 4 weeks. If the patient changed from manic episode to depressive episode, according to the patient’s condition, fluoxetine capsule (Manufacturer: Jiangsu Hengrui Medicine Co., Ltd, State Drug License H20030138, specification 20 mg) was selected to be used, 20 mg per day, orally, with a course of treatment for 4 weeks. In addition, patients were given routine nursing measures such as health education, life guidance and psychological intervention.

The intervention group received affective intervention in addition to conventional pharmacotherapy, not as a replacement. All patients in both groups received pharmacological treatment based on national guidelines for bipolar disorder, including titrated doses of lithium carbonate and quetiapine. Lithium levels were monitored weekly to ensure therapeutic range (0.6–1.2 mmol/L). Affective intervention in the experimental group was introduced after partial remission of acute manic symptoms, usually within 3 to 5 days of hospitalization, when patients demonstrated sufficient behavioral stability and compliance.

The intervention group implemented a 4-week affective intervention based on the control group, and the specific interventions for the intervention group are shown in Table 1.

**Table 1:** Intervention group specific interventions.

Sports event	Specific measures
Cognitive restructuring	Through the guidance of a professional psychotherapist, the patient’s negative automatic thoughts, such as “I can’t do anything about it” and “No one understands me,” are identified and gradually replaced with more positive and rational thought patterns, such as “I can handle challenges,” “I have a support system,” and “I have a support system." “I have a support system.” Each session included a 20-minute in-depth conversation to explore the patient’s thinking patterns and to help him or her identify and challenge irrational beliefs. The 40min practice exercise after each session consisted of recording negative thinking that occurred in daily life and attempting to adjust it. 1x/h, 2x/wk.
Behavioral activation	Individualized activity plans are developed according to the patient’s interests and abilities, such as participating in community activities, engaging in physical exercise or learning new skills. Weekly records of activity participation and patient feedback are kept to help patients gradually increase their social participation and activity levels and improve life satisfaction.
Coping strategies training	Teach deep breathing techniques to help patients calm down quickly when they are agitated by taking slow, deep, long breaths. Gradually relax the muscles in all parts of the body, starting from the toes to the head, to help patients relax when they are tense. Simulate social situations to teach patients effective communication skills and social etiquette to help them improve their interpersonal relationships. 1x/h, 2x/wk.
Positive thought meditation	Teach patients to focus on their breathing and feel every inhalation and exhalation to help them improve their awareness and emotional regulation. Instruct patients to gradually focus on various parts of the body, perceive the state of relaxation and tension, and promote physical and mental relaxation. Through slow, focused walking, feel the contact between the feet and the ground, to help patients improve their awareness and concentration. 1x/h, 2x/wk.
Emotion recognition and expression training	Teach patients to recognize different emotion words, such as “happy,” “angry,” “sad,” to help them express their emotions more accurately. Through simulated situations, help patients to practice emotional expression and improve their ability to manage emotions in real life. 1x/h, 2x/week
Psycho-pedagogical courses	To explain the basic theories and mechanisms of emotion and help patients understand the process of emotion generation and regulation. Teach the skills of emotion recognition and expression to help patients express their emotions rationally in different situations. Teach strategies to cope with emotional fluctuations, such as deep breathing, positive thinking meditation, emotion recognition and expression training, etc, to help patients cope effectively with emotional fluctuations. 1x/h, 1x/week

Throughout the course of treatment, patients were closely observed for affective and cognitive changes, and their manic symptoms and quality of life changes were assessed regularly. The assessment tools include Young Mania Rating Scale and Quality of Life Scale (SF-36), which are assessed before treatment, 2 weeks after treatment and at the end of treatment, respectively, in order to have a comprehensive understanding of the efficacy of the affective intervention. Attention was paid to observing and recording patients’ adverse reactions and compliance during treatment to ensure the safety and efficacy of the treatment. To account for inter-individual differences in lithium metabolism, serum lithium levels were monitored weekly during the study, and maintained within a therapeutic range of 0.6 to 1.0 mmol/L through dose adjustment. This ensured consistency in drug exposure across patients receiving the same nominal dose.

### 2.3. Observation indicators

The Bech-Rafaelsen Mania Symptom Severity Scale (BRMS)^[14]^ was used to evaluate the patients’ manic symptoms, which consisted of 11 items, including elevated mood, increased speech, increased self-assessment, decreased sleep, euphoria, racing thoughts, running thoughts, impulsive behaviors, hypersexuality, aggressive behaviors, inattentiveness, and social disorders. Each item was scored according to the severity of symptoms, with 4 points for each item, 0 corresponding to low performance, 4 corresponding to high performance, and the scores of each item were added together to give a total score of 0 to 44. The higher the score, the more severe the manic symptoms.

Patients’ quality of life was evaluated using The Short Form (36) Health Survey (SF-36),^[15]^ which contains 36 items covering physical functioning, role physical, Bodily Pain (Bodily Pain, BP), general health, Vitality (VT), SF, role emotional, and mental health. Each domain is scored through specific questions. Scores for each domain range from 0 to 100, with higher scores indicating better overall health and quality of life.

The Insight and Treatment Attitudes Questionnaire (ITAQ)^[16]^ was used to evaluate the patient’s cognitive situation, which contains 4 aspects: awareness of both the disease, awareness of hospitalization, awareness of attitude towards taking medication, and awareness of disease recurrence and help-seeking, with each item scored according to the patient’s response. The total score ranges from 0 to 22. The higher the total score, the more positive the patient’s knowledge of his/her disease and attitude toward treatment.

### 2.4. Statistical analysis

SPSS27.0 (Chicago) statistical software was used to statistically analyze the data of this study. Count data were expressed as percentage (%) and the results were tested by χ² test. Measurement information was expressed by ($\bar{x}\pm s$), and the results were tested by t-test, with *P* <.05 indicating that the difference was statistically significant.

## 3. Results

### 3.1. Comparison of baseline information between the 2 groups

There was no significant difference between the baseline data of the 2 groups (*P *>.05). See Table 2.

**Table 2:** Comparison of baseline information between the 2 groups.

Groups	Number of examples	Gender (cases)		Age (year)	Duration of illness (year)	BMI (kg/m^2^)
		Male	Women			
Control subjects	38	17	21	27.54 ± 5.70	4.85 ± 2.31	24.47 ± 2.51
Intervention group	38	20	18	27.83 ± 5.05	4.52 ± 2.99	24.29 ± 2.72
χ2/*t*-value	–	0.474		0.235	0.538	0.300
*P*-value	–	.491		.815	.592	.765

BMI = body mass index.

### 3.2. Comparison of manic symptom scores between the 2 groups of patients

**Figure 1.**
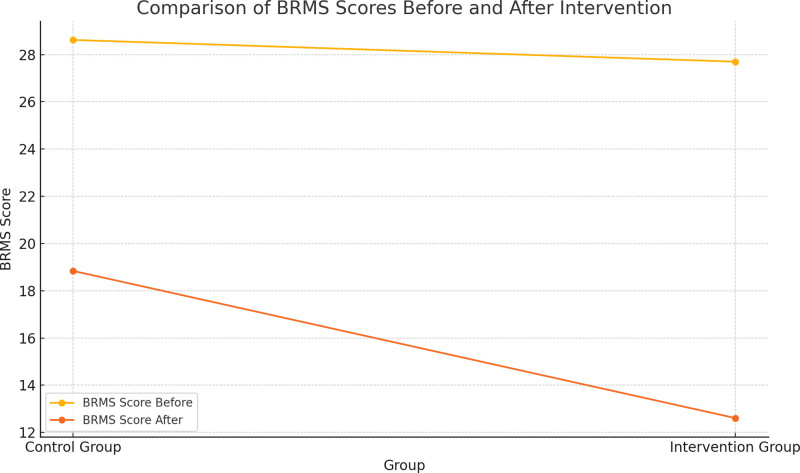
Line graph of manic symptom scores for both groups of patients.

Before the intervention, the BRMS scores of the 2 groups were compared (*P *>.05), after the intervention, the BRMS scores of both groups were lower than before the intervention, and the intervention group was lower than the control group (*P *<.05). See Table 3 and Figure 1.

**Table 3:** Comparison of manic symptom scores between the 2 groups ($\bar{x}\pm \;s$).

Groups	Number of examples	BRMS score	
		Pre-intervention	Post-intervention
Control subjects	38	28.62 ± 3.60	18.84 ± 2.92*
Intervention group	38	27.70 ± 3.71	12.61 ± 2.33*
*t*-value	–	1.097	10.280
*P*-value	–	.276	<.001

BRMS = Bech–Rafaelsen Mania Scale.

* *P* <.05 compared to pre-intervention in this group.

### 3.3. Comparison of SF-36 scores between the 2 groups

**Figure 2.**
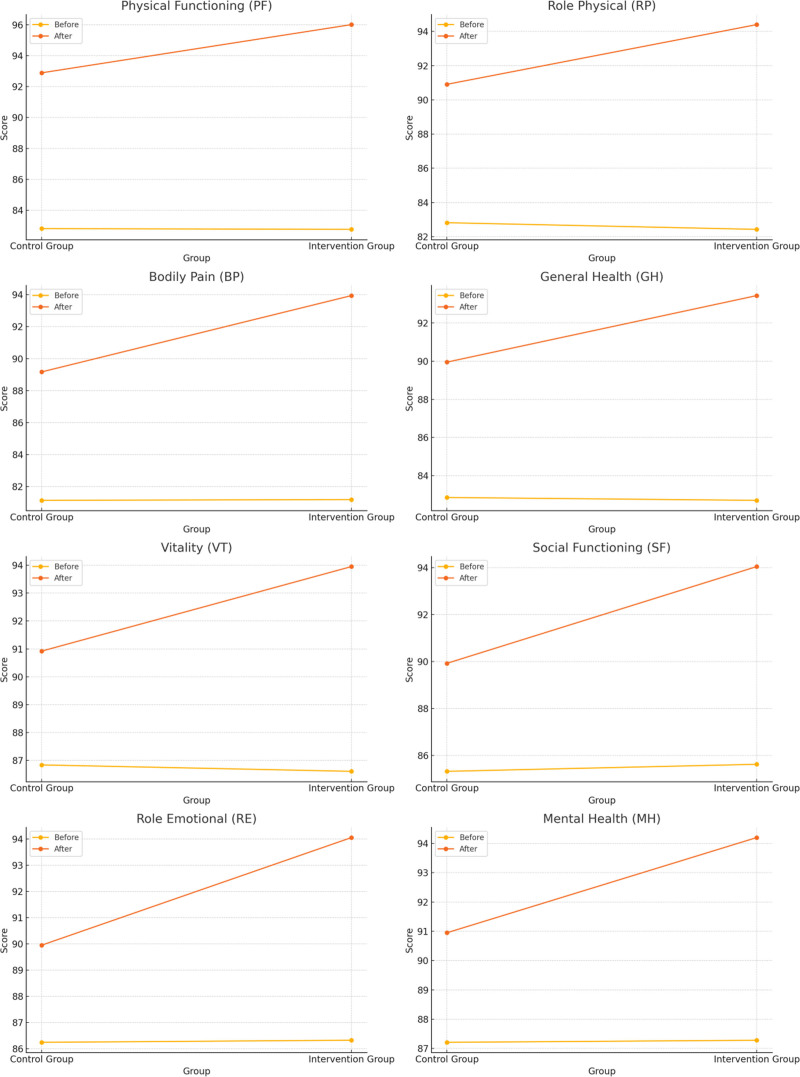
Line graph of SF-36 scores for both groups. SF-36 = health survey.

Before the intervention, the SF-36 scores of the 2 groups were compared *(P *>.05), and after the intervention, the SF-36 scores of both groups were higher than before the intervention, and the intervention group was higher than the control group (*P *<.05). See Table 4 and Figure 2.

**Table 4:** Comparison of SF-36 scores between the 2 groups ($\bar{x}\pm \;s$).

groups	PF			RP		BP		GH	
	pre-intervention	post-intervention		pre-intervention	post-intervention	pre-intervention	post-intervention	pre-intervention	post-intervention
control subjects	82.81 ± 3.46		92.89 ± 3.11*	82.82 ± 4.11	90.91 ± 4.60*	81.14 ± 3.16	89.17 ± 3.32*	82.85 ± 3.29	89.95 ± 3.65*
intervention group	82.76 ± 3.50		96.01 ± 2.77*	82.43 ± 4.06	94.40 ± 4.73*	81.19 ± 3.25	93.94 ± 3.38*	82.70 ± 3.22	93.44 ± 4.02*
*t*-value	0.063		4.618	0.416	3.261	0.068	6.206	0.201	3.962
*P*-value	.950		<.001	.679	.002	.946	<.001	.841	<.001

groups	VT			SF		RE		MH	
	pre-intervention	post-intervention		pre-intervention	post-intervention	pre-intervention	post-intervention	pre-intervention	post-intervention
control subjects	86.84 ± 3.40		90.92 ± 3.52*	85.32 ± 3.21	89.92 ± 3.12*	86.24 ± 2.95	89.95 ± 3.28*	87.21 ± 2.71	90.95 ± 2.33*
intervention group	86.61 ± 3.39		93.95 ± 3.66*	85.62 ± 3.17	94.04 ± 3.36*	86.32 ± 3.05	94.06 ± 3.35*	87.28 ± 2.88	94.20 ± 2.37*
*t*-value	0.295		3.678	0.410	5.539	0.116	5.404	0.109	6.028
*P*-value	.769		<.001	.683	<.001	.908	<.001	.913	<.001

BP = bodily pain, GH = general health, MH = mental health, PF = physical functioning, RE = role emotional, RP = role physical, SF = social functioning, SF-36 = health survey, VT = vitality.

* *P* <.05 compared to pre-intervention in this group.

### 3.4. Comparison of ITAQ scores between the 2 groups of patients

**Figure 3.**
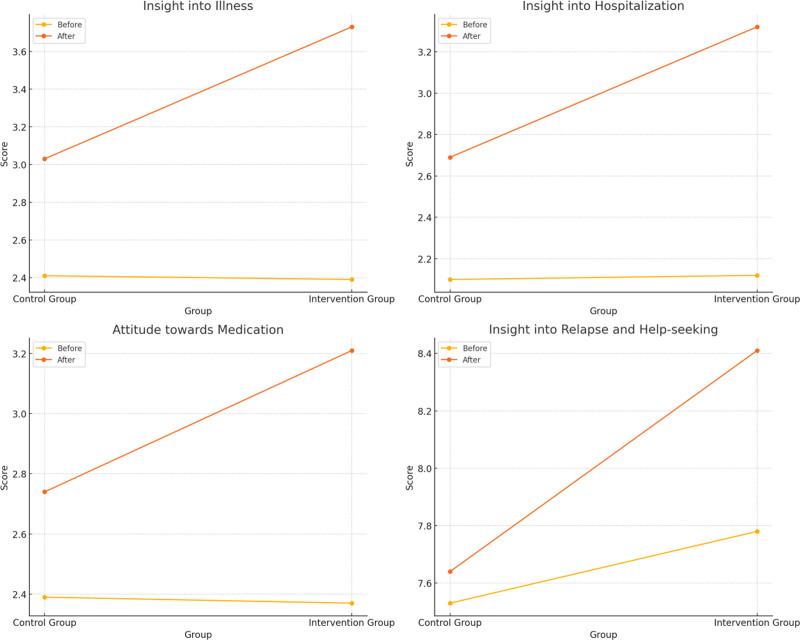
Line graph of ITAQ scores for both groups of patients. ITAQ = insight and treatment attitudes questionnaire.

Before the intervention, the ITAQ scores of the 2 groups were compared (*P *>.05), after the intervention, the ITAQ scores of both groups were higher than before the intervention, and the intervention group was higher than the control group (*P *<.05). See Table 5 and Figure 3.

**Table 5:** Comparison of ITAQ scores between the 2 groups ($\bar{x}\pm \;s$).

groups	Disease awareness			Awareness of hospitalization		Awareness of attitudes towards taking medication		Relapse and help-seeking	
	pre-intervention	post-intervention		pre-intervention	post-intervention	pre-intervention	post-intervention	pre-intervention	post-intervention
control subjects	2.41 ± 1.06		3.03 ± 1.04*	2.10 ± 1.27	2.69 ± 1.30*	2.39 ± 1.12	2.74 ± 0.82	7.53 ± 1.12	7.64 ± 1.51
intervention group	2.39 ± 1.05		3.73 ± 1.01*	2.12 ± 1.24	3.32 ± 1.02*	2.37 ± 1.06	3.21 ± 0.72*	7.78 ± 1.09	8.41 ± 1.55*
*t*-value	0.083		2.976	0.069	2.350	0.080	2.655	0.986	2.194
*P*-value	.934		.004	.945	.021	.936	.010	.327	.031

ITAQ = insight and treatment attitudes questionnaire.

* *P* <.05 compared to pre-intervention in this group.

## 4. Discussion

With the in-depth research on bipolar disorder, the role of affective interventions in the regulation of manic symptoms and quality of life has been gradually emphasized.^[17]^ Bipolar disorder is a psychological disorder that seriously affects an individual’s cognitive function and emotion regulation ability, and the pathogenesis is complex, involving multiple factors such as genetics, neurobiology and environmental factors.^[18,19]^ In recent years, with the improvement of the mental health service system, more and more studies have begun to focus on the positive impact of emotional intervention on patients with bipolar disorder. Affective intervention is a therapeutic approach aimed at improving the emotional state and quality of life of patients through emotional education, emotional support, and emotional regulation techniques. Mardani et al^[20]^ showed that affective intervention can effectively reduce the manic symptom scores and improve the emotional stability of patients with bipolar disorder. The main mechanisms of affective interventions include enhancing patients’ affective sensory perception through affective education, improving patients’ social support through affective support, and helping patients better cope with emotional fluctuations through affective regulation techniques.

In the treatment of bipolar disorder, medication mainly controls manic and depressive symptoms by regulating neurotransmitter levels,^[21]^ whereas affective interventions radically improve patients’ quality of life by improving their emotional regulation and cognitive functioning. Marone et al^[22]^ pointed out that affective interventions can significantly improve quality of life scores of patients with bipolar disorder, reduce relapse rates, and reduce hospitalization time. Affective intervention focuses on the psychological and social factors of the patients and improves the symptoms and quality of life of the patients at multiple levels through comprehensive intervention strategies. Cognitive impairment is a common problem in patients with bipolar disorder, which is mainly characterized by inattention, memory loss and executive dysfunction.^[23–25]^ Affective interventions can effectively improve cognitive functioning through cognitive restructuring and emotion regulation techniques. Astami et al^[26,27]^ found that patients with BAD who received affective interventions performed significantly better than those who did not receive interventions on cognitive functioning tests, with particularly significant improvements in attention and executive functioning. Patients with bipolar disorder often face social isolation and interpersonal tensions due to fluctuating emotions and impaired cognitive functioning.^[28]^ Emotional interventions help patients rebuild social support networks, improve interpersonal relationships, and enhance SF through emotional support and social skills training. Moot et al^[29]^ demonstrated that emotional interventions can significantly enhance the social competence and self-efficacy of patients with BAD, which can promote the recovery of their SF. Emotional intervention significantly enhances patients’ psychological resilience and social adaptability by establishing and strengthening their social support system, providing emotional support, information support and practical help.

The results of this study showed that affective interventions can significantly improve the quality of life and cognitive function of patients with bipolar disorder. The reason for this is that affective intervention effectively reduces patients’ manic symptoms by improving their emotion regulation ability and cognitive function.^[30]^ Enhanced emotion regulation and improved cognitive functioning keep their spirit in a relatively stable state, thus reducing the symptoms of mood swings and hyperarousal. Through cognitive-behavioral therapy, patients are able to learn more effective emotion management and coping strategies, which reduces the negative effects of emotional loss of control. Through enhanced interaction and communication between family members and patients, patients are able to gain more emotional support and understanding, which helps them stabilize their emotions and improve their quality of life. This social support can not only alleviate the patient’s sense of loneliness and helplessness but also promote the patient’s recovery through positive interactions.^[31,32]^ Emotional interventions can alleviate manic symptoms through physiological feedback mechanisms, and through targeted emotion regulation training, patients can learn to better recognize and regulate their emotions, thus reducing physical discomfort and mental stress caused by mood swings. Michel et al^[33,34]^ showed that sleep disorders are one of the important features of manic symptoms. Emotional interventions enable patients to obtain more adequate and high-quality rest by improving their sleep patterns, thus further contributing to the stabilization of their mood and overall quality of life.

Limitations of this study include a small sample size and a short study period that did not adequately reflect long-term effects. The study population was mainly from a single healthcare organization, and there was selection bias, which may limit the generalizability of the results. The study did not collect detailed sociodemographic variables (e.g., marital status, employment, physical activity levels), which may influence quality of life measures. Although inpatient hospitalization provided some standardization, these factors remain potential confounders. In addition, the specific implementation method and frequency of affective interventions may have different effects due to individual differences, and further research is needed to optimize the intervention protocol. By recognizing these limitations, we can assess the study results more objectively and enhance their credibility.

In summary, affective intervention has significant efficacy in improving manic symptoms, cognitive function and quality of life in patients with bipolar disorder. Through the comprehensive application of emotion education, emotion support and emotion regulation techniques, affective intervention provides a proven therapeutic program for patients with bipolar disorder, which is worthy of further promotion and application in clinical practice. Future studies should further explore the specific mechanisms and optimal intervention strategies of affective intervention, with a view to providing a more scientific and comprehensive treatment program for patients with bipolar disorder.

## Author contributions

**Conceptualization:** Bingwei Zhang.

**Data curation:** Chao Li.

**Formal analysis:** Chao Li.

**Funding acquisition:** Chao Li.

**Investigation:** Chao Li, Suilin Jia.

**Methodology:** Suilin Jia.

**Project administration:** Suilin Jia.

**Resources:** Suilin Jia.

**Software:** Suilin Jia, Guangdong Chen.

**Supervision:** Guangdong Chen.

**Validation:** Guangdong Chen.

**Visualization:** Bingwei Zhang, Guangdong Chen.

**Writing – original draft:** Bingwei Zhang.

**Writing – review & editing:** Bingwei Zhang.
